# 2,6-Bis(bromo­meth­yl)pyridine

**DOI:** 10.1107/S1600536813032364

**Published:** 2013-12-04

**Authors:** Olesea Cuzan, Tatiana Straistari, Constantin Turta, Marius Réglier

**Affiliations:** a Institute of Chemistry of the Academy of Sciences of Moldova, 3 Academiei Street, Chisinau MD-2028, Republic of Moldova; b Aix Marseille Université, ISM2 UMR 7313, Campus de St. Jérôme, Av. Escadrille Normandie Niemen, 13397 Marseille Cedex 20, France

## Abstract

In the title mol­ecule, C_7_H_7_Br_2_N, the C—Br vectors of the bromo­methyl groups extend to opposite sides of the pyridine ring and are oriented nearly perpendicular to its plane. In the crystal, the mol­ecules related by a *c*-glide-plane operation are arranged into stacks along the *c* axis, with centroid–centroid distances between neighboring aromatic rings of 3.778 (2) Å. A short Br⋯Br contact of 3.6025 (11) Å is observed within a pair of inversion-related mol­ecules.

## Related literature   

For the isomorphous crystal structure of 2,6-bis­(chloro­meth­yl)pyridine, see: Betz *et al.* (2011 ▶). For the synthesis of the title compound, see: Dioury *et al.* (2009 ▶).
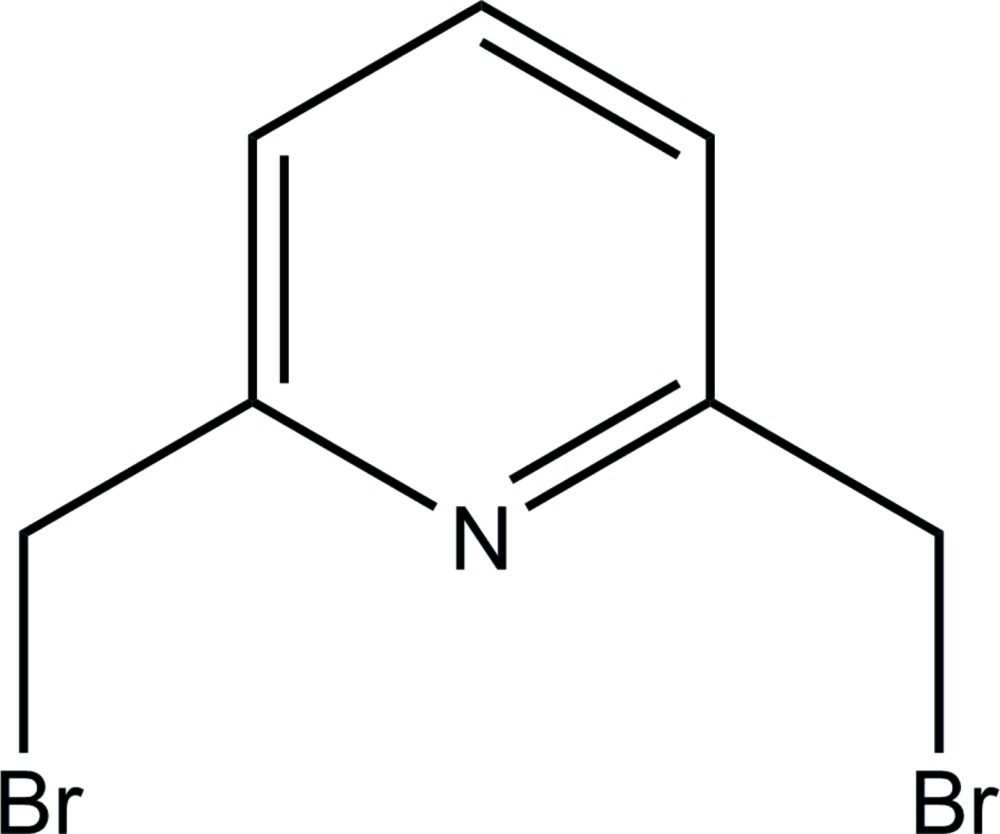


## Experimental   

### 

#### Crystal data  

C_7_H_7_Br_2_N
*M* *_r_* = 264.96
Monoclinic, 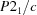

*a* = 9.2955 (19) Å
*b* = 12.980 (3) Å
*c* = 7.5288 (15) Å
β = 110.75 (3)°
*V* = 849.5 (3) Å^3^
*Z* = 4
Mo *K*α radiation
μ = 9.47 mm^−1^
*T* = 293 K
0.28 × 0.26 × 0.12 mm

#### Data collection  

Nonius KappaCCD diffractometer
Absorption correction: multi-scan (*SORTAV*; Blessing, 1995 ▶) *T*_min_ = 0.088, *T*_max_ = 0.321
10219 measured reflections
2480 independent reflections
1923 reflections with *I* > 2σ(*I*)
*R*_int_ = 0.048

#### Refinement  

*R*[*F* ^2^ > 2σ(*F* ^2^)] = 0.044
*wR*(*F* ^2^) = 0.119
*S* = 1.04
2480 reflections
92 parameters
H-atom parameters constrained
Δρ_max_ = 0.99 e Å^−3^
Δρ_min_ = −1.06 e Å^−3^

### 

Data collection: *COLLECT* (Nonius, 1998 ▶); cell refinement: *DENZO*/*SCALEPACK* (Otwinowski & Minor, 1997 ▶); data reduction: *DENZO*/*SCALEPACK*; program(s) used to solve structure: *SHELXS97* (Sheldrick, 2008 ▶); program(s) used to refine structure: *SHELXL97* (Sheldrick, 2008 ▶); molecular graphics: *Mercury* (Macrae *et al.*, 2006 ▶) and *XP* in *SHELXTL* (Sheldrick, 2008 ▶); software used to prepare material for publication: *enCIFer* (Allen *et al.*, 2004 ▶) and *PLATON* (Spek, 2009 ▶).

## Supplementary Material

Crystal structure: contains datablock(s) I. DOI: 10.1107/S1600536813032364/gk2596sup1.cif

Structure factors: contains datablock(s) I. DOI: 10.1107/S1600536813032364/gk2596Isup2.hkl

Supporting information file. DOI: 10.1107/S1600536813032364/gk2596Isup3.cml

Additional supporting information:  crystallographic information; 3D view; checkCIF report

## Figures and Tables

**Fig. 1.**
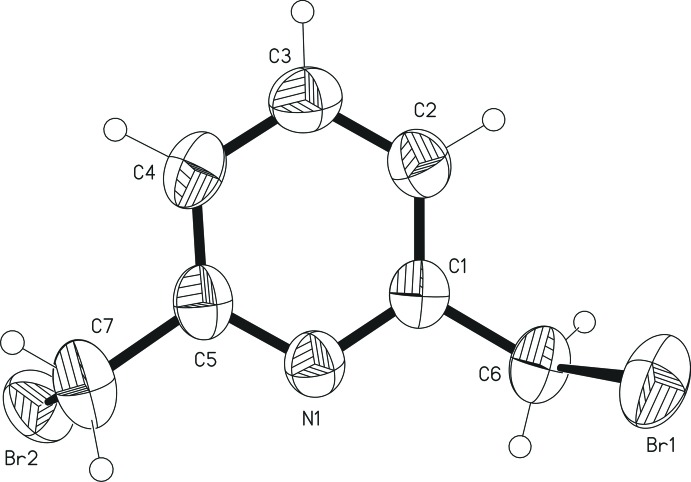
The molecular structure of the title compound. Displacement ellipsoids are drawn at the 50% probability level. H atoms are presented as small spheres of arbitrary radius.

**Fig. 2.**
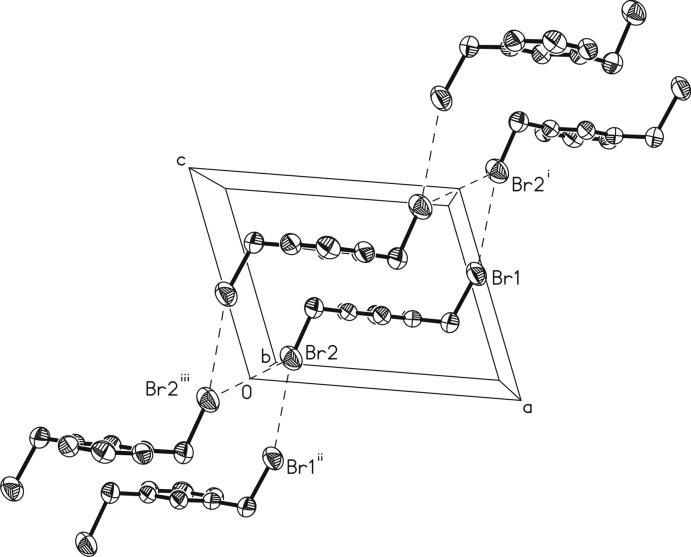
Intermolecular Br···Br contacts (dashed lines) in the title compound. Symmetry codes: (i) 1 + x, y, 1 + z; (ii) -1 + x, y, -1 + z; (iii) -x, -y, -z. H atoms have been omitted for clarity.

## supplementary crystallographic information

### 1. Comment

2,6-Bis(bromomethyl)pyridine is a well known organic compound which serves as a precursor in various organic reactions leading to formation of a large range of pyridine derivatives. Additionally, the presence of N-donor atom enables coordination of metal ions by this molecule. Due to conformational flexibility of the bromomethyl arms, the title compound has been used as a starting material for the synthesis of macrocycles (Dioury *et al.*, 2009).

Recently, the crystal structure of the Cl analogue of the title compound - 2,6-bis(chloromethyl)pyridine - has been reported and the two compounds form an isomorphous pair.

In the crystal, π-π stacking interaction between aromatic rings of molecules forming stacks along the *c* axis are observed. The distance between the centroids of the stacked pyridine rings is 3.778 (2) Å (symmetry code: *x*, 1/2 - *y*, -1/2 + *z).*

The bromine atoms are situated at both sides of the pyridine plane and bromomethyl arms are pointing at approximately opposite directions. In the crystal, there are two different Br···Br contacts of type I. The contact longer than the sum of van der Waals radii [Br2···Br1^i^ 3.7051 (11) Å; symmetry code: (i) 1 + *x*, *y*, 1 + *z*] links the molecules into infinite chains along [1 0 1] whereas the other one [Br2···Br2^iii^ 3.6025 (11) Å; symmetry code: (iii) -*x*, -*y*, -*z*] is formed between neighbouring chains.

### 2. Experimental

The compund was obtained according to previously reported procedure from the 2,6-bis(hydroxymethyl)pyridine (Dioury *et al.*, 2009). Crystals suitable for the X-ray diffraction study were obtained by recrystallization from diethylether at room temperature.

### 3. Refinement

The H atoms were positioned geometrically and refined using a riding model with C—H = 0.95–0.99 Å and with *U*_iso_(H) = 1.2 times *U*_eq_(C).

### Crystal data

**Table:**

C_7_H_7_Br_2_N	*F*(000) = 504
*M**_r_* = 264.96	*D*_x_ = 2.072 Mg m^−^^3^
Monoclinic, *P*2_1_/*c*	Melting point = 358–360 K
Hall symbol: -P 2ybc	Mo *K*α radiation, λ = 0.71073 Å
*a* = 9.2955 (19) Å	Cell parameters from 10219 reflections
*b* = 12.980 (3) Å	θ = 2.8–30.4°
*c* = 7.5288 (15) Å	µ = 9.47 mm^−^^1^
β = 110.75 (3)°	*T* = 293 K
*V* = 849.5 (3) Å^3^	Platelet, colourless
*Z* = 4	0.28 × 0.26 × 0.12 mm

### Data collection

**Table:**

Nonius KappaCCD diffractometer	2480 independent reflections
Radiation source: fine-focus sealed tube	1923 reflections with *I* > 2σ(*I*)
Graphite monochromator	*R*_int_ = 0.048
ω and Phi scan	θ_max_ = 30.4°, θ_min_ = 2.8°
Absorption correction: multi-scan (*SORTAV*; Blessing, 1995)	*h* = −11→13
*T*_min_ = 0.088, *T*_max_ = 0.321	*k* = −17→14
10219 measured reflections	*l* = −10→8

### Refinement

**Table:**

Refinement on *F*^2^	Secondary atom site location: difference Fourier map
Least-squares matrix: full	Hydrogen site location: inferred from neighbouring sites
*R*[*F*^2^ > 2σ(*F*^2^)] = 0.044	H-atom parameters constrained
*wR*(*F*^2^) = 0.119	*w* = 1/[σ^2^(*F*_o_^2^) + (0.0467*P*)^2^ + 1.3504*P*] where *P* = (*F*_o_^2^ + 2*F*_c_^2^)/3
*S* = 1.04	(Δ/σ)_max_ < 0.001
2480 reflections	Δρ_max_ = 0.99 e Å^−^^3^
92 parameters	Δρ_min_ = −1.06 e Å^−^^3^
0 restraints	Extinction correction: *SHELXL97* (Sheldrick, 2008), Fc^*^=kFc[1+0.001xFc^2^λ^3^/sin(2θ)]^-1/4^
Primary atom site location: structure-invariant direct methods	Extinction coefficient: 0.036 (3)

### Special details

**Table:**

Geometry. All e.s.d.'s (except the e.s.d. in the dihedral angle between two l.s. planes) are estimated using the full covariance matrix. The cell e.s.d.'s are taken into account individually in the estimation of e.s.d.'s in distances, angles and torsion angles; correlations between e.s.d.'s in cell parameters are only used when they are defined by crystal symmetry. An approximate (isotropic) treatment of cell e.s.d.'s is used for estimating e.s.d.'s involving l.s. planes.
Refinement. Refinement of *F*^2^ against ALL reflections. The weighted *R*-factor *wR* and goodness of fit *S* are based on *F*^2^, conventional *R*-factors *R* are based on *F*, with *F* set to zero for negative *F*^2^. The threshold expression of *F*^2^ > σ(*F*^2^) is used only for calculating *R*-factors(gt) *etc*. and is not relevant to the choice of reflections for refinement. *R*-factors based on *F*^2^ are statistically about twice as large as those based on *F*, and *R*-factors based on ALL data will be even larger.

### Fractional atomic coordinates and isotropic or equivalent isotropic displacement parameters (Å^2^)

**Table:**

	*x*	*y*	*z*	*U*_iso_*/*U*_eq_	
Br1	0.97970 (5)	0.13488 (4)	0.59690 (7)	0.0698 (2)	
Br2	0.17000 (5)	0.07677 (4)	0.10894 (7)	0.0696 (2)	
N1	0.5656 (3)	0.1307 (2)	0.3525 (4)	0.0461 (6)	
C1	0.6810 (4)	0.1899 (3)	0.3462 (5)	0.0447 (7)	
C2	0.6751 (4)	0.2963 (3)	0.3423 (5)	0.0509 (8)	
H2	0.7580	0.3347	0.3371	0.061*	
C3	0.5438 (5)	0.3448 (3)	0.3464 (6)	0.0560 (9)	
H3	0.5369	0.4162	0.3454	0.067*	
C4	0.4230 (4)	0.2844 (3)	0.3521 (5)	0.0535 (9)	
H4	0.3329	0.3148	0.3536	0.064*	
C5	0.4380 (4)	0.1781 (3)	0.3554 (5)	0.0479 (8)	
C6	0.8218 (4)	0.1350 (4)	0.3439 (6)	0.0559 (9)	
H6A	0.8610	0.1688	0.2555	0.067*	
H6B	0.7957	0.0646	0.3015	0.067*	
C7	0.3117 (5)	0.1099 (4)	0.3646 (6)	0.0646 (11)	
H7A	0.2560	0.1441	0.4350	0.078*	
H7B	0.3552	0.0468	0.4310	0.078*	

### Atomic displacement parameters (Å^2^)

**Table:**

	*U*^11^	*U*^22^	*U*^33^	*U*^12^	*U*^13^	*U*^23^
Br1	0.0465 (3)	0.0867 (4)	0.0672 (3)	0.01422 (19)	0.00885 (19)	−0.0045 (2)
Br2	0.0558 (3)	0.0711 (3)	0.0710 (3)	−0.01709 (19)	0.0091 (2)	0.0004 (2)
N1	0.0412 (15)	0.0523 (16)	0.0429 (15)	0.0000 (12)	0.0127 (12)	0.0034 (12)
C1	0.0419 (16)	0.0556 (19)	0.0357 (15)	0.0006 (14)	0.0127 (13)	0.0011 (13)
C2	0.0466 (18)	0.056 (2)	0.0481 (19)	−0.0040 (15)	0.0141 (15)	0.0016 (15)
C3	0.057 (2)	0.052 (2)	0.052 (2)	0.0039 (16)	0.0115 (17)	−0.0016 (16)
C4	0.0417 (17)	0.070 (2)	0.0460 (18)	0.0105 (16)	0.0117 (14)	−0.0023 (16)
C5	0.0382 (16)	0.065 (2)	0.0374 (16)	−0.0015 (14)	0.0100 (13)	0.0030 (15)
C6	0.0458 (19)	0.072 (3)	0.052 (2)	0.0069 (17)	0.0199 (16)	0.0008 (17)
C7	0.048 (2)	0.092 (3)	0.055 (2)	−0.010 (2)	0.0190 (18)	0.008 (2)

### Geometric parameters (Å, º)

**Table:**

Br1—C6	1.950 (4)	C3—H3	0.9300
Br2—C7	1.956 (4)	C4—C5	1.387 (6)
N1—C1	1.334 (4)	C4—H4	0.9300
N1—C5	1.343 (5)	C5—C7	1.491 (5)
C1—C2	1.382 (6)	C6—H6A	0.9700
C1—C6	1.496 (5)	C6—H6B	0.9700
C2—C3	1.383 (6)	C7—H7A	0.9700
C2—H2	0.9300	C7—H7B	0.9700
C3—C4	1.382 (6)		
			
C1—N1—C5	117.5 (3)	N1—C5—C7	116.3 (4)
N1—C1—C2	123.4 (3)	C4—C5—C7	121.1 (4)
N1—C1—C6	116.3 (3)	C1—C6—Br1	110.4 (3)
C2—C1—C6	120.3 (4)	C1—C6—H6A	109.6
C1—C2—C3	118.8 (4)	Br1—C6—H6A	109.6
C1—C2—H2	120.6	C1—C6—H6B	109.6
C3—C2—H2	120.6	Br1—C6—H6B	109.6
C4—C3—C2	118.4 (4)	H6A—C6—H6B	108.1
C4—C3—H3	120.8	C5—C7—Br2	110.6 (3)
C2—C3—H3	120.8	C5—C7—H7A	109.5
C3—C4—C5	119.2 (3)	Br2—C7—H7A	109.5
C3—C4—H4	120.4	C5—C7—H7B	109.5
C5—C4—H4	120.4	Br2—C7—H7B	109.5
N1—C5—C4	122.6 (3)	H7A—C7—H7B	108.1
			
C5—N1—C1—C2	0.0 (5)	C1—N1—C5—C7	−179.4 (3)
C5—N1—C1—C6	179.7 (3)	C3—C4—C5—N1	−0.3 (6)
N1—C1—C2—C3	0.4 (6)	C3—C4—C5—C7	179.0 (4)
C6—C1—C2—C3	−179.4 (3)	N1—C1—C6—Br1	−100.1 (3)
C1—C2—C3—C4	−0.7 (6)	C2—C1—C6—Br1	79.7 (4)
C2—C3—C4—C5	0.6 (6)	N1—C5—C7—Br2	−91.3 (4)
C1—N1—C5—C4	0.0 (5)	C4—C5—C7—Br2	89.4 (4)
